# Molecular evidence on the presence of *Schistosoma japonicum* infection in snails along the Yangtze River, 2015–2019

**DOI:** 10.1186/s40249-022-00995-9

**Published:** 2022-06-18

**Authors:** Yin-Long Li, Hui Dang, Su-Ying Guo, Li-Juan Zhang, Yun Feng, Song-Jun Ding, Xiao-Wei Shan, Guang-Ping Li, Min Yuan, Jing Xu, Shi-Zhu Li

**Affiliations:** 1 National Institute of Parasitic Diseases, Chinese Center for Disease Control and Prevention (Chinese Center for Tropical Diseases Research), Shanghai, 200025 People’s Republic of China; 2 NHC Key Laboratory of Parasite and Vector Biology, Shanghai, 200025 People’s Republic of China; 3 WHO Collaborating Centre for Tropical Diseases, Shanghai, 200025 People’s Republic of China; 4 National Center for International Research on Tropical Diseases, Shanghai, 200025 People’s Republic of China; 5 Jiangsu Provincial Institute of Schistosomiasis Control, Wuxi, Jiangsu Province 214064 People’s Republic of China; 6 Anhui Provincial Institute of Schistosomiasis Control, Hefei, Anhui Province 230061 People’s Republic of China; 7 Hubei Provincial Institute of Schistosomiasis Control, Hubei Center for Disease Control, Wuhan, Hubei Province 430079 People’s Republic of China; 8 Hunan Provincial Institute of Schistosomiasis Control, Hunan Province 414000, Yueyang, People’s Republic of China; 9 Jiangxi Provincial Institute of Parasitic Disease, Nanchang, Jiangxi Province 330006 People’s Republic of China

**Keywords:** *Schistosoma japonicum*, Dissection, Loop-mediated isothermal amplification, The Yangtze River, Transmission risk, Low endemic area

## Abstract

**Background:**

Due to sustained control activities, the prevalence of *Schistosoma japonicum* infection in humans, livestock and snails has decreased significantly in P. R. China, and the target has shifted from control to elimination according to the Outline of Healthy China 2030 Plan. Applying highly sensitive methods to explore the presence of *S. japonicum* infection in its intermediate host will benefit to assess the endemicity or verify the transmission interruption of schistosomiasis accurately. The aim of this study was to access the presence of *S. japonicum* infection by a loop-mediated isothermal amplification (LAMP) method through a 5-year longitudinal study in five lake provinces along the Yangtze River.

**Methods:**

Based on previous epidemiological data, about 260 villages with potential transmission risk of schistosomiasis were selected from endemic counties in five lake provinces along the Yangtze River annually from 2015 to 2019. Snail surveys were conducted in selected villages by systematic sampling method and/or environmental sampling method each year. All live snails collected from field were detected by microscopic dissection method, and then about one third of them were detected by LAMP method to assess the presence of *S. japonicum* infection with a single blind manner. The infection rate and nucleic acid positive rate of schistosomes in snails, as well as the indicators reflecting the snails’ distribution were calculated and analyzed. Fisher's exact test was used to examine any change of positive rate of schistosomes in snails over time.

**Results:**

The 5-year survey covered 94,241 ha of environment with 33,897 ha of snail habitats detected accumulatively. Totally 145.3 ha new snail habitats and 524.4 ha re-emergent snail habitats were found during 2015–2019. The percentage of frames with snails decreased from 5.93% [45,152/761,492, 95% confidence intervals (*CI*): 5.88–5.98%] in 2015 to 5.25% (30,947/589,583, 95% *CI*: 5.19–5.31%) in 2019, while the mean density of living snails fluctuated but presented a downward trend generally from 0.20 snails/frame (155,622/761,492, 95% *CI*: 0.17–0.37) in 2015 to 0.13 snails/frame (76,144/589,583, 95% *CI*: 0.11–0.39) in 2019. A total of 555,393 live snails were collected, none of them was positive by dissection method. Totally 17 pooling snail samples were determined as positives by LAMP method among 8716 pooling samples with 174,822 of living snails, distributed in 12 villages of Hubei, Hunan, Jiangxi and Anhui provinces. The annual average positive rate was 0.41% (95% *CI*: 0.13–0.69%) in 2015, 0% in 2016, 0.36% (95% *CI*: 0.09–0.63%) in 2017, 0.05% (95% *CI*: 0–0.16%) in 2018, 0.05% (95% *CI*: 0–0.15%) in 2019, respectively, presenting a downward trend from 2015 to 2019 with statistical significance (*χ*^2^ = 11.64, *P* < 0.05).

**Conclusions:**

The results suggest that *S. japonicum* infection still persisted in nature along the Yangtze River and traditional techniques might underestimate the prevalence of schistosomiasis in its intermediate hosts. Exploring and integrating molecular techniques into national surveillance programme could improve the sensitivity of surveillance system and provide guidance on taking actions against schistosomiasis.

**Graphical Abstract:**

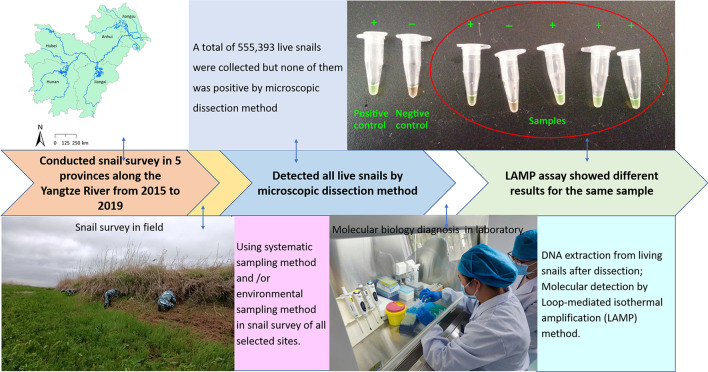

**Supplementary Information:**

The online version contains supplementary material available at 10.1186/s40249-022-00995-9.

## Background

Schistosomiasis (bilharzia), as one of the neglected tropical diseases, is a vector-borne disease caused by one of six species of *Schistosoma* parasite that affects at least 230 million people in parts of the Middle East, South America, Southeast Asia and Africa [1, 2]. In Asia, schistosomiasis japonica, caused by *S. japonicum*, is prevalent among human beings and over 40 species of mammals, and distributes in the south of China, the Philippines and small regions of Indonesia [3, 4]. With over two thousand years of endemic history, schistosomiasis japonica used to be distributed in 12 provinces along the Yangtze River of China with an estimated 11.6 million cases and 1.2 million infected bovines in the 1950s [5–7]. Through nearly 70-year hard work, China has made tremendous strides in prevention and control of schistosomiasis especially after implementing the integrated control strategy at the beginning of the new millennium [8], which made the prevalence and infection intensity of *S. japonicum* decreased dramatically [9]. Transmission control (prevalence in human beings and livestock both less than 1% in all endemic villages) was reached nationwide by 2015 [10]. In 2016, a national strategic plan of Healthy China 2030 was released by the central government to improve health of Chinese people and a goal was set to eliminate schistosomiasis in all endemic counties by 2030 [11].

However, challenge exists to interrupt the transmission of schistosomiasis entirely in China due to extensive snail habitats, multiple mammals including wildlife served as infection resources, unhealthy behavior of residents, frequently occurring floods, increasing population mobilization etc. [12, 13]. Particularly, opposite to the decreased trends of prevalence in human beings and livestock, the snail breeding area increased from 3562.8 million m^2^ in 2015 to 3623.68 million m^2^ in 2019 due to the flooding occurred in 2016, which were mainly distributed in five lake provinces along the Yangtze River [14–16]. As various studies have proved that the rebound of snail burden always occurred earlier than an increased incidence of schistosomiasis, understanding the snail breeding trend and detecting the pathogen of schistosomes in snails could provide useful and accurate insight into ongoing transmission of schistosomiasis, guide the implementation of interventions and verify elimination of transmission [17].

Conventionally, the infection of schistosome in snails are based on collecting snails from field followed by microscopical examination of schistosome cercariae or shedding cercariae by exposed snails to light [18]. These methods require considerable time, efforts and expertise in identification of schistosome cercariae or sporocysts. In addition, their shortcomings such as missing early or light infections became obvious with the decrease of prevalence and infection intensity in endemic areas [19]. More sensitive and rapid tools are needed emergently for schistosomiasis surveillance and quick response [20, 21]. Molecular xenomonitoring is an alternative approach to detect pathogens in their vectors, which had been reported for monitoring the transmission of parasitic diseases, such as malaria, filariasis, sleeping sickness as well as schistosomiasis, etc. [22–24]. Polymerase-chain reaction (PCR), nested-PCR, quantitative PCR (q-PCR) and isothermal amplification techniques, etc. were widely used as the tools of molecular xenomonitoring, presenting advantages over traditional parasitological methods to detect early and light infections in vectors. Loop-mediated isothermal amplification (LAMP), a novel isothermal amplification method developed by Notomi in 2000 [25], is considered to have great potential to be used for field settings due to its various advantages such as high sensitivity, rapidity and ease of use in laboratories without expensive equipment. Additionally, the sample pooling strategy could be integrated to improve resource efficiency and made LAMP more applicable to be used in poor settings. Although several studies explored the feasibility and applicability of LAMP technique for detecting the schistosomes in *Oncomelania hupensis*, large scale application of LAMP technique for snail detection is less reported.

To assess and map the persistence of *S. japonicum* in natural environment and provide information for programme managers, we conducted a large scale field survey in five lake provinces of China with potential risk of schistosomiasis transmission from 2015 to 2019, and a well-documented LAMP technique improved by our labs was used to detect the infection of *S. japonicum* in snails. The results may offer evidence as an attempt of the application of the novel sensitive approach and provide the reference for stakeholders to achieve the future goal of schistosomiasis elimination in China.

## Methods

### Study sites

Based on epidemiological data of previous years, one administrative village from each endemic county which supposed having potential risk of schistosomiasis transmission in Hubei, Hunan, Jiangxi, Anhui and Jiangsu provinces was selected annually during 2015–2019. These villages had at least one of the following features: (1) used to be high-prevalence settings and the environment of snail habitats didn’t change essentially; (2) appeared a trend of rebound of snail breeding area or density of living snails, or detected infected snails or developing new snail habitats, or a relative high infection rate or antibody positive rate in humans or livestock according to the latest survey; (3) snail breeding habitats changed greatly due to natural disasters such as floods or earthquakes, or the construction of large-scale water conservancy, transportation and other projects; (4) had large-scale population migration or movement which may lead to occurrence of schistosomiasis.

### Snail survey

**Fig. 1:**
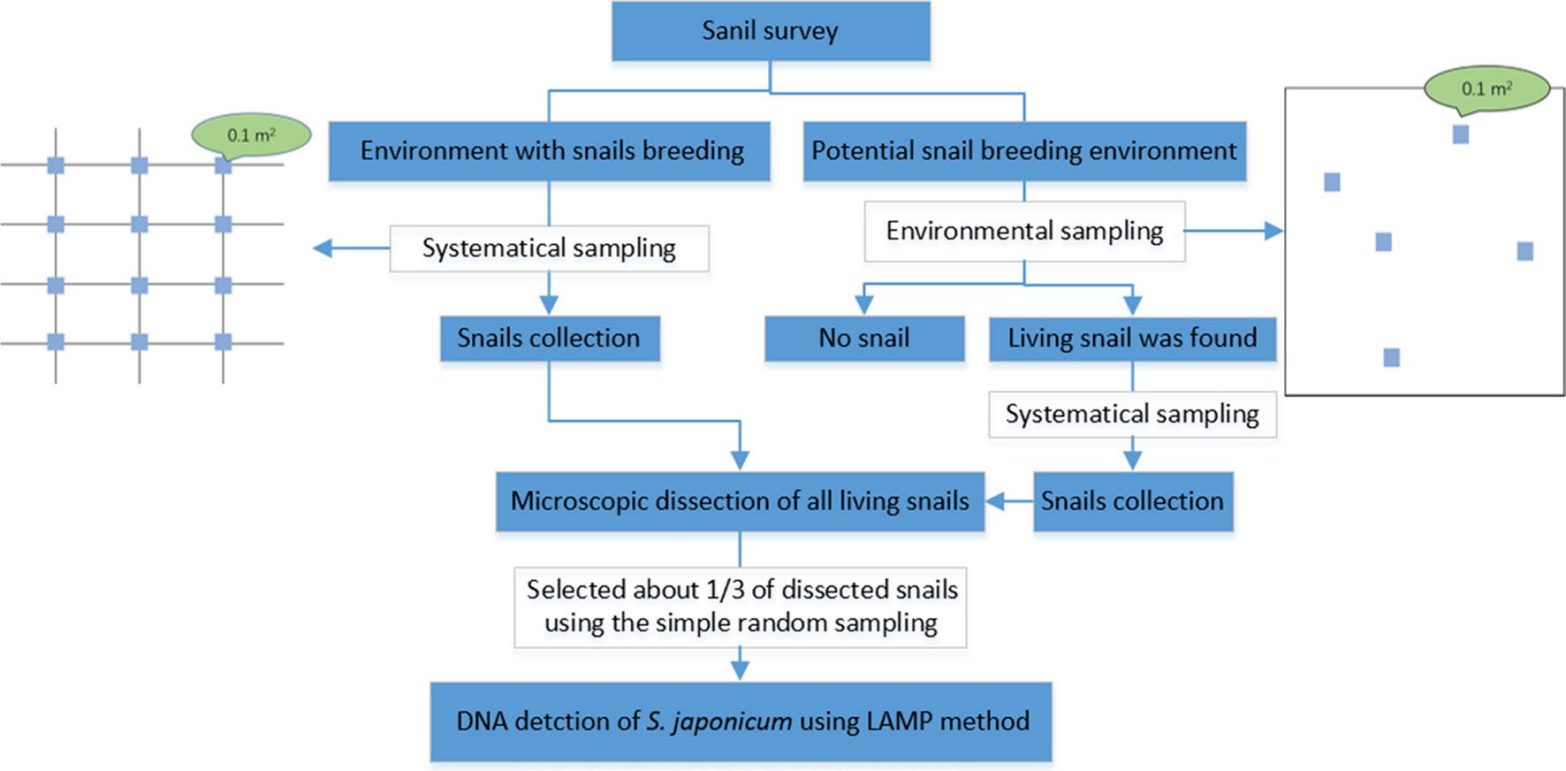
The general flow chart of our study. *LAMP* loop-mediated isothermal amplification

Snail survey was conducted in spring annually during 2015–2019 using systematic sampling and/or environmental sampling methods. A frame with area of 0.1 m^2^ was used and set to investigate snails [26]. Systematic sampling method was used directly in environment with snails breeding according to the previous survey [27]. In settings neighbored with snail habitats and suspected having snails distributed potentially, environmental sampling method would be used firstly and followed by systematic sampling method once living snail was found [27]. The distance between any two frames and the number of frames set were determined by the ecological feature or areas of surveyed environment. All snails within the frames were collected, counted and brought to the laboratory to be examined. See Fig. 1.

### Microscopic dissection of snails

All *Oncomelania hupensis* snails collected from field and identified based on shell morphology were washed and placed in a dish or porcelain cup filled with dechlorinated water at room temperature observation for 2–3 h. Snails presenting any open movement, extension of soft tissue, contraction after acupuncture were determined as alive. The living snails were placed on a slide and kept a certain distance, and then crushed gently by another thick slide. One drop of dechlorinated water was added to each snail and the slide was moved to microscopy for examination. The snail was determined as infected one if the sporocyst and/or cercariae of *S. japonicum* were found in the soft tissue of dissected snails.

### DNA extraction from snails

The soft tissue of dissected snails was collected separately and labeled according to environment. Every 10 snail tissues from the same environment after dissection were placed in a clean 2.0 ml tube and DNA extraction was performed using a commercial DNA extraction kit (Tiangen Biotech Co. Ltd., Beijing, China) in accordance with the manufacturer's instructions. Every 5 tubes of exacted DNA from the same environment were mixed as one sample for LAMP detection.

### LAMP detection

To detect the DNA of *S. japonicum*, genomic DNA extracted from the pooled snails was used as the template for the LAMP assay using the primers target to the mitochondrial 28S rRNA gene of *S. japonicum* [19]. The reaction was carried out in a final volume of 25 µl as follows: 2.0 µl of genomic DNA template, 12.5 µl of 2 × LAMP reaction buffer, 1.0 µl of amplification primer mixture, 7.5 µl of sterilized deionized water, 1.0 µl of Bst DNA polymerase and 1.0 µl of chromogenic reagent. In each amplification reaction, genomic DNA extracted from adult worms of *S. japonicum* and sterilized deionized water were used to replace the extracted DNA template to set positive and negative controls. All reaction tubes were incubated at 65 ℃ for 60–90 min, and then observed with naked eyes. The tube with liquid presented as green was considered as positive while the yellow–brown tube as negative.

### Data management and statistical analysis

Annual data obtained were collected and entered into computer by Microsoft Excel software, version 2013 (Microsoft Office, CA, USA) in a standardized manner. The snail burden was measured by the mean density of living snails, the percentage of frames with living snails and the infection rate of schistosomes in snails determined by microscopic dissection method. The area reflecting the distribution of snails was expressed as length (meters) multiplied by width (meters). The length and width were determined based on the largest distance between two frames which found live snails or infected snails in the surveyed environments. LAMP results were expressed as the percentages of positive pools and surveys sites. Fisher's exact test was used to examine the change of positive rates determined by LAMP method among different years. Confidence intervals (*CI*) were calculated using standard formulas based on the binomial distribution and significance was assigned as *P* value less than 0.05.

## Results

### General information of selected sites

From 2015 to 2019, about 260 villages were selected from five provinces along the Yangtze River for snail survey in each year. The number of villages in Anhui, Jiangxi, Hubei and Hunan maintained in 51, 39, 63 and 41, respectively each year, while the villages selected in Jiangsu Province varied slightly from 59 to 67 affected by the change of administrative division.

### Distribution of snails

During 2015–2019, snail investigation was conducted in 94,241 ha of areas cumulatively. Among 33,897 ha of areas confirmed breeding snails, totally 145.3 ha of areas were new developed snail habitats and 524.4 ha of areas were re-emergent snail habitats. During 2015–2019, totally 3,213,493 frames were set for snail’s investigation, and 555,393 living snails were found in 199,415 set frames. The percentage of frames with living snails ranged from 5.25% (95% *CI*: 5.19–5.31%) to 7.87% (95% *CI*: 7.80–7.94%), while the mean density of living snails presented a downward trend from 2015 to 2019 (Table 1).

**Table 1:** Summary of snail survey during 2015 to 2019

Year	Area surveyed (ha)	Area infested with snails (ha)	Area of new snail habitats (ha)	Area with snails reemerged in previous habitats (ha)	No. frames surveyed	No. frames with snail	No. dissected living snails	No. infected snails	Percentage of frames with living snails, % (95% *CI*)	Mean density of living snails, (number of snails per frame, pf) (95% *CI*)
2015	18,043	7271	3.5	19.7	761,492	45,152	155,622	0	5.93 (5.88–5.98)	0.20 (0.17–0.37)
2016	17,832	6810	136.6	74	597,849	47,045	132,968	0	7.87 (7.80–7.94)	0.22 (0.18–0.46)
2017	19,241	6682	0.2	88.9	680,497	41,452	105,151	0	6.09 (6.03–6.15)	0.15 (0.16–0.44)
2018	19,303	6297	5	112	584,072	34,819	85,508	0	5.96 (5.90–6.02)	0.16 (0.16–0.27)
2019	19,822	6837	0	229.8	589,583	30,947	76,144	0	5.25 (5.19–5.31)	0.13 (0.11–0.39)
Total	94,241	33,897	145.3	524.4	3,213,493	199,415	555,393	0	–	–

*ha* hectare; 95% *CI* 95% confidence intervals, *pf* per frame (0.11 m^2^), – means not applicable

**Fig. 2:**
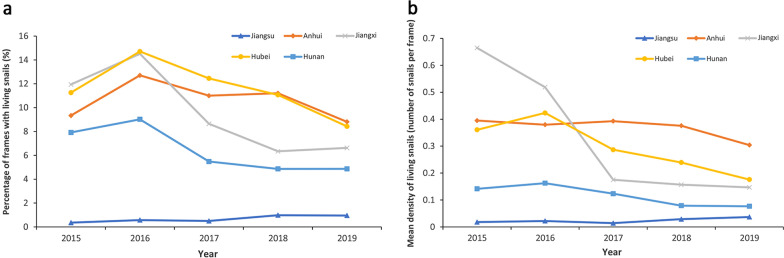
The change trends of snail burden in 5 province, 2015 to 2019. **a** Percentage of frames with living snails. **b** Mean density of living snails

The percentage of frames with living snails and mean density of living snails based on provincial level were presented in Fig. 2. Among the five provinces, Jiangsu province always presented the lowest percentage of frames with living snails in the range of 0.36% (95% *CI*: 0.34–0.38%)–0.98% (95% *CI*: 0.94–1.02%), and mean density of living snails varied from 0.01 snail per frame (pf) (95% *CI*: 0–0.75 pf) to 0.04 pf (95% *CI*: 0.02–0.11 pf). Jiangxi and Hubei kept the top level of snail burden during the five years with the percentage of frames with living snails in the range of 6.34% (95% *CI*: 6.11–6.56%)–14.52% *(*95% *CI*: 14.09–14.94%), 8.43% (95% *CI*: 8.31–8.56%)–14.71% (95% *CI*: 14.54–14.87%) and the mean density of living snails in the range of 0.15 pf (95% *CI*: 0.01–0.49 pf)–0.66 pf (95% *CI*: 0–1.36 pf), 0.18 pf (95% *CI*: 0.10–0.26 pf)–0.42 pf (95% *CI*: 0.11–0.35 pf), respectively (see Additional file 1). On the whole, the snail burden in five provinces showed a slow downward trend but fluctuated in 2016 due to the flooding occurred that year.

Among the selected sites where systematic sampling using for the snail survey, 4 sites have the higher percentage of frames with living snails (> 75%), 1 in Hubei in 2015, 1 in Anhui in 2016, and 1 in Jiangsu in 2016 and 2017, respectively. In addition, 3 sites have the higher mean density of frame with living snail (> 10 pf), 1 in Jiangsu in 2016 and 2017, respectively, and 1 in Anhui in 2019. In the sites without living snails collected, Jiangsu always kept a relatively high proportion every year, up to 78.57% (44/56) in 2015.

### Snail detection by microscopic dissection and LAMP

From 2015 to 2019, 555,393 living snails were detected by dissection method but no positive sample was found (Table 1). Among dissected snails, soft tissue of 174,822 snails (accounted for 31.48%) were randomly selected for DNA extraction and 8716 pooled nucleic acid samples were obtained. Totally, 17 positive mixed samples were detected in Hubei, Hunan Jiangxi and Anhui province by LAMP method. The average positive rate of pooled sample ranged from 0 to 0.41% (95% *CI*: 0.13–0.69%) annually, presenting a decreased trend (Table 2) with statistical significance detected by Fisher's exact test (*χ*^2^ = 11.64, *P* = 0.01).

**Table 2:** The results of LAMP for detecting *S. japonicum* in snails from 2015 to 2019

Year	No. live snails detected by LAMP method	No. nucleic acid mixed samples (tubes)	No. positive nucleic acid mixed samples	Positive rate %, (95% *CI*)
2015	39,302	1971	8	0.41 (0.13–0.69)
2016	16,184	981	0	0
2017	34,712	1948	7	0.36 (0.09–0.63)
2018	43,659	1847	1	0.05 (0–0.16)
2019	40,965	1969	1	0.05 (0–0.15)
Total	174,822	8716	17	–

*LAMP* loop-mediated isothermal amplification, *CI* confidence intervals, – means not applicable

The 17 positive mixed samples by LAMP method were distributed in 12 villages of Hubei, Hunan, Jiangxi and Anhui province. Among of them, 6 were collected from 5 villages of Hunan province, 5 from 4 villages of Anhui province, 5 from 2 villages of Jiangxi Province while the left one from one village of Hubei Province. Among all positive sites, 83.33% of the positive sites (10/12) had a percentage of frames with living snails of less than 25%, and the mean density of living snails was less than 1 snail per frame. The largest snail area of positive site was about 165 ha located in Jiangxi Province in 2015, and the smallest one is about 0.14 ha located in Anhui Province in 2015.

## Discussion

In 2020, WHO published a new road map to guide action against NTDs during the decade 2021–2030. The road map targets the elimination of schistosomiasis as a public health problem by 2030. With decades of sustained efforts [28], China has made remarkable achievements in schistosomiasis control and put forward the goal of eliminating schistosomiasis in all counties by 2030 [11]. However, it is difficult to eliminate schistosomiasis due to the zoonotic nature of *S. japonicum* and the amphibious characteristics of snails [29–31], so it is necessary to strengthen surveillance to provide guidance for schistosomiasis control and elimination.

To better understand the epidemic trend of schistosomiasis and assess the effectiveness of control measures, China had conducted systematic surveillance on schistosomiasis since the end of twentieth century [32]. As schistosomiasis is a vector-borne disease, understanding the distribution of *Oncomelania hupensis* and its infection status would benefit to assess the prevalence and transmission of schistosomiasis [33, 34], or serve as a residual infection marker to a certain extent [35]. Thus, survey on snails was listed a main content of schistosomiasis surveillance activities together with survey on human beings and livestock in China. According to the recent report of Xu et al. the infection rate of schistosome in snails had been decreased from 0.26% in 2005 to 0 in 2015 by traditional dissection method. However, infection in animals or human beings still occurred in some settings where no infected snails detected, indicating the shortcomings of traditional techniques to identify the infection of schistosomes in snails became apparent and more sensitive methods are needed to estimate the endemic situation of schistosomiasis and identify the transmission risk accurately in low endemic area [21, 36]. Molecular xenomonitoring using advanced and more sensitive molecular techniques, has been proved an effective alternative of tradition methods to detect the pathogens in intermediate hosts of vector-borne diseases [39]. Being a technique which had been assessed through laboratory and field assessment, LAMP is rapid, specific, and convenient in detection of *S. japonicum* infection in snails with isothermal DNA polymerase, and can be applied as a new molecular diagnosis in the field in endemic areas [35].

In our study, a five-year study was conducted to detect the presence of *S. japonicum* infection of snails in villages with potential transmission of schistosomiasis selected from five provinces along the Yangtze River. From 2015 to 2019, no infected snail was detected by microscopy dissection method in five provinces, indicating the success of schistosomiasis control obtained in China. When detecting the snails by LAMP method, totally 17 positive mixed snail samples were found. Several reasons can be explained for the inconsistence of two methods: (1) Snails have been infected at an early stage and cannot be detected by microscopy, but can be detected by LAMP method due to its high sensitivity [35]; (2) The microscope inspector missed the inspection because of the low infection of *S. japonicum*. (3) The LAMP results may be all false positives. Base on good performance in laboratory and field application of snail nucleic acid detection of LAMP method in our previous work [19], the third reason can be excluded. Thus, the result obtained by LAMP method indicated that the transmission of schistosomiasis is still continued along the Yangtze River in China but kept a very low level.

In addition, measurement of the changes of snail burden (percentage of frames with living snails, mean density of living snails) and mapping the geographic information of snail distribution based on snail survey can contribute to guide the snail control activities precisely. In our study, the snail burden in five provinces showed a slow downward trend but fluctuated in 2016 due to the flooding occurred that year. Relevant studies showed that flood disaster could cause snail diffusion and increase the area and density of snails [38]. Meanwhile, the geographical distribution of all snails’ habitats is almost in five provinces along the Yangtze River, with over 95% of total snail areas in China [39], which is consistent with schistosome-endemic areas [27]. So, it is a vital intervention for interrupting the life cycle of *S. japonicum* in snail control to reduce the transmission risk in China [27, 33, 40]. In areas where schistosomiasis transmission has been interrupted, it is necessary to monitor the snails in existing and historical snail habitats and access the transmission risk of schistosomiasis by using advanced techniques [41, 42].

Several previous reports have suggested that LAMP is useful for the detection of the infections in pathogen-carrying vectors [43, 44], and it also showed good performance of sensitive in accessing the presence of *S. japonicum* infection of snails in our study. It means that the current prevalence of schistosomiasis may be underestimated, and may be a huge hidden danger to achieve the goal of eliminating schistosomiasis in the future. This study also demonstrates that LAMP is an appropriate molecular xenomonitoring tool in assessing the presence of *S. japonicum* infection in low endemic areas due to its being higher sensitivity in snail diagnosis, and may play an important role in the elimination of schistosomiasis of China in the future. To reach the goal of schistosomiasis elimination, further integration of interventions, multisector collaboration, sensitive and effective surveillance are also needed to strengthen [40, 45].

Several limitations of our study should be noted. First, the villages with potential risk of schistosomiasis were not collected randomly while only one-third of snails dissected were examined by LAMP method randomly based on the consideration of the cost, which may cause the bias of estimation of the infection status of snails. Second, as the pooling strategy was used, the LAMP method can identify the environment with transmission risk but couldn’t estimate the accurate infection rate of snails. Further studies should be conducted to explore how to estimate the infection rate of schistosome in snails or transmission intensity in combination with modelling methods.

## Conclusions

Five years of longitudinal research proved the success of schistosomiasis control in China and indicated that the current prevalence of schistosomiasis may be underestimated due to the insensitivity of traditional methods. LAMP technique is a suitable molecular xenomonitoring tool for snail detection in low-endemic areas of schistosomiasis because of its higher sensitivity, and could be used to verify whether the transmission of schistosomiasis had been interrupted in endemic regions. To achieve the ultimate goal of schistosomiasis elimination in P.R. China, surveillance-response system should be improved, particularly to strengthen the surveillance on intermediate snail host of *S. japonicum*.

## Supplementary Information

**Additional file 1: Table S1.** Percentage of frames with living snails in each province from 2015 to 2019. **Table S2.** The mean density of living snails in 5 provinces from 2015 to 2019.

## Data Availability

All data generated or analyzed during this study are confidentially kept at the National Institute of Parasitic Disease, Chinese Center for Disease Control and Prevention. The datasets are available from the corresponding author on a reasonable request.

## Abbreviations

ha: Hectare
*CI*: Confidence intervals
pf: Per frame (0.11 m^2^)
LAMP: Loop-mediated isothermal amplification
WHO: World Health Organization
P.R.China: People’s Republic of China
