# Supplementary material for: Molecular evidence on the presence of Schistosoma japonicum infection in snails along the Yangtze River, 2015–2019
Source: Infect Dis Poverty. 2022 Jun 18;11:70. doi: 10.1186/s40249-022-00995-9 (PMC9206329; doi:10.1186/s40249-022-00995-9)
Supplement: Supplementary file 1 — Additional file 1: Table S1. Percentage of frames with living snails in each province from 2015 to 2019. Table S2. The mean density of living snails in 5 provinces from 2015 to 2019. [file 40249_2022_995_MOESM1_ESM.pdf]

Supplementary files

Table S1. Percentage of frames with living snails in each province from 2015 to 2019

Year	Items	Jiangsu	Anhui	Jiangxi	Hubei	Hunan
2015	No. frames surveyed	330,594	88,566	38,272	211,234	92,826
	No. frames with snail	1,175	8,268	4,571	23,790	7,348
	Percentage of frames with living snails, % (95% *CI*)	0.36(0.34−0.38)	9.34(9.14−9.53)	11.94(11.62−12.27)	11.26(11.13−11.40)	7.92(7.74−8.09)
2016	No. frames surveyed	248,134	70,752	26,584	176,021	76,358
	No. frames with snail	1,419	8,989	3,859	25,889	6,889
	Percentage of frames with living snails, % (95% *CI*)	0.57(0.54−0.60)	12.70(12.46−12.95)	14.52(14.09−14.94)	14.71(14.54−14.87)	9.02(8.82−9.23)
2017	No. frames surveyed	289,545	74,176	45,536	187,178	84,062
	No. frames with snail	1,437	8,161	3,936	23,310	4,608
	Percentage of frames with living snails, % (95% *CI*)	0.50(0.47−0.52)	11.00(10.78−11.23)	8.64(8.39−8.90)	12.45(12.30−12.60)	5.48(5.33−5.64)
2018	No. frames surveyed	229,642	71,367	46,728	162,984	73,351
	No. frames with snail	2,253	7,998	2,962	18,039	3,567
	Percentage of frames with living snails, % (95% *CI*)	0.98(0.94−1.02)	11.21(10.98−11.44)	6.34(6.11−6.56)	11.07(10.92−11.22)	4.86(4.71−5.02)
2019	No. frames surveyed	209,916	79,574	41,130	185,297	73,666
	No. frames with snail	2,001	7,012	2,725	15,624	3,585
	Percentage of frames with living snails, % (95% *CI*)	0.95(0.95−0.99)	8.81(8.62−9.00)	6.63(6.39−6.87)	8.43(8.31−8.56)	4.87(4.71−5.02)

Table S2. The mean density of living snails in 5 provinces from 2015 to 2019

Year	Items	Jiangsu	Anhui	Jiangxi	Hubei	Hunan
2015	No. frames surveyed	330,594	88,566	38,272	211,234	92,826
	No. living snails	5,932	34,971	25,447	76,153	13,119
	Mean density of living snails, (Pf) (95% *CI*)	0.02(0−0.10)	0.39(0.23−0.86)	0.66(0−1.36)	0.36(0.11−0.34)	0.14(0.048−0.27)
2016	No. frames surveyed	248,134	70,752	26,584	176,021	76,358
	No. living snails	5,419	26,853	13,793	74,504	12,399
	Mean density of living snails, (Pf) (95% *CI*)	0.02(0−0.94)	0.38(0.31−0.93)	0.52(0−0.85)	0.42(0.11−0.35)	0.16(0.075−0.35)
2017	No. frames surveyed	289,545	74,176	45,536	187,178	84,062
	No. living snails	4,043	29,134	7,965	53,643	10,366
	Mean density of living snails, (Pf) (95% *CI*)	0.01(0−0.75)	0.39(0.35−0.93)	0.17(0.06−0.43)	0.29(0.13−0.28)	0.12(0.052−0.24)
2018	No. frames surveyed	229,642	71,367	46,728	162,984	73,351
	No. living snails	6,593	26,811	7,325	38,984	5,795
	Mean density of living snails, (Pf) (95% *CI*)	0.03(0.05−0.24)	0.38(0.30−0.66)	0.16(0.07−0.44)	0.24(0.12−0.28)	0.08(0.05−0.19)
2019	No. frames surveyed	209,916	79,574	41,130	185,297	73,666
	No. living snails	7,694	24,190	6,031	32,588	5,641
	Mean density of living snails, (Pf) (95% *CI*)	0.04(0.02−0.11)	0.30(0.04−1.35)	0.15(0.01−0.49)	0.18(0.10−0.26)	0.08(0.05−1.80)
